# Inhibition of PDE4B ameliorates cognitive defects in the model of alcoholic dementia in 3xTg-AD mice via PDE4B/cAMP/PKA signaling

**DOI:** 10.1093/ijnp/pyaf009

**Published:** 2025-02-08

**Authors:** Rongzhen Sun, Mei Han, Yuanyuan Lin, Shengyao Ma, Huan Tu, Xueliang Yang, Fang Zhang, Han-Ting Zhang

**Affiliations:** Department of Pharmacology, Qingdao University School of Pharmacy, Qingdao, China; Department of Pharmacology, Qingdao University School of Pharmacy, Qingdao, China; Department of Pharmacology, Qingdao University School of Pharmacy, Qingdao, China; Department of Pharmacology, Qingdao University School of Pharmacy, Qingdao, China; Department of Pharmacology, Qingdao University School of Pharmacy, Qingdao, China; Department of Pharmacology, Qingdao University School of Pharmacy, Qingdao, China; Department of Pharmacology, Qingdao University School of Pharmacy, Qingdao, China; Department of Pharmacology, Qingdao University School of Pharmacy, Qingdao, China

**Keywords:** alcoholic dementia, phosphodiesterase-4B, 3xTg-AD mice, cognitive deficits

## Abstract

**Background:**

Chronic, heavy alcohol use may lead to permanent brain damage, cognitive impairment, and dementia. One of the most serious consequences is alcoholic dementia (AlD). Phosphodiesterase-4 (PDE4) inhibitors have been shown to exhibit beneficial effects on cognition deficits and alcoholism. However, it is not known whether PDE4 inhibitors can be used to treat AlD. A33, a relatively selective PDE4B inhibitor, is absent of the emetic effect associated with PDE4D. The effect of A33 on memory and cognition in AlD remains unclear.

**Methods:**

We investigated the effects of A33 and the PDE4 inhibitor rolipram on memory and cognition using an AlD animal model, that is, APP/PS1/Tau mice drinking alcohol in the 2-bottle choice test, with or without A33 or rolipram treatment for 3 weeks. The animal groups were compared in behavioral tests related to learning and memory. Neurochemical measures were conducted to explore the underlying mechanism of A33.

**Results:**

Compared to wild-type controls, AlD mice showed impairments of learning ability and memory in the behavior tests; this was attenuated by treatment of rolipram or A33. In addition, administration of rolipram or A33 in AlD mice further alleviated neuropathological alterations in the hippocampus, including Aβ expression and deposition; rolipram or A33 also decreased the levels of inflammatory cytokines, including interleukin-1β (IL-1β), interleukin-6 (IL-6), and tumor necrosis factor-α (TNF-α), as well as nuclear factor kappa-B (NF-κB). Further, rolipram or A33 decreased the activation of microglia while increased cyclic adenosine monophosphate (cAMP) levels in the hippocampus of AlD mice.

**Conclusions:**

These results revealed that the alleviation of the cognitive impairment of AlD in APP/PS1/Tau triple transgenic mice by rolipram or A33 was linked to the action of the PDE4B/cAMP/PKA signaling pathway. A33 can be a promising therapeutic agent for AlD-related cognitive dysfunction.

**Significance Statement**

Chronic, heavy alcohol use may lead to permanent brain damage, cognitive impairment, and dementia. One of the most serious consequences is alcoholic dementia (AlD). Rolipram, a nonselective phosphodiesterase-4 (PDE4) inhibitor, has been shown to treat cognitive impairment, depression, anxiety, and alcohol dependence. However, the side effects such as emesis and vomiting hinder its clinical application. A33 is a relatively selective PDE4B inhibitor that has been shown to reduce alcohol consumption and alcohol preference in mice, with potent anti-inflammatory effects and without the side effect of emesis. In AlD mice, administration of rolipram or A33 reduced the neuropathological changes in the hippocampus, including Aβ expression and deposition. It also reduced inflammatory factors and hippocampal microglia activation, and increased the level of cAMP in AlD mice. These results suggest that inhibition of PDE4 or its PDE4B subtype attenuates cognitive impairment related to alcoholic dementia via activation of the PDE4B/cAMP/PKA signaling pathway.

## INTRODUCTION

Alcohol dependence or alcoholism is a mental disease that seriously endangers human health. According to the World Health Organization, more than 2 billion people worldwide drink alcohol; among them, 140 million are alcohol dependent. Alcoholic dementia (AlD) is a serious consequence of alcohol dependence and abuse.^1^ Chronic, excessive alcohol consumption leads to memory decline and cognitive impairment, accompanied by personality changes, tremors, delirium, and even spasms.^2^ Alcohol dependence has been shown to contribute to the development of Alzheimer’s disease (AD), which can be worsened by heavy alcohol drinking depending on the duration of alcohol dependence.^3^ Considering the high prevalence of AD among all forms of dementia and the maturity of 3xTg-AD mice as a transgenic model of AD, 3xTg-AD mice have been used to set up the model of AlD.^4^ Therefore, we employed 3xTg-AD mice exposed to alcohol using the 2-bottle choice paradigm to establish a mouse model AlD. The pathogenesis of AlD, the most severe state of alcoholism, remains unclear. Microglia, which have been thought to be involved in AlD, can sense and respond to central nervous system (CNS) injury promptly.^5^ Microglial priming is considered to lead to self-perpetuating downstream expression of inflammatory cytokines and chemokines.^6,7^ In alcohol use disorders (AUDs), microglial activation can be pathogenic, leading to neuroinflammation, neuronal loss, synapse loss, tissue damage, and behavioral changes.^4,8,9^

A growing body of evidence suggests a link between alcohol and neuroinflammation.^10,11^ Alcoholism and chronic use of alcohol affect the immune system, causing microglial responses that release proinflammatory cytokines. Microglial activation has been demonstrated in mouse models of alcohol dependence.^12,13^ Consistent with this, clinical studies have also found that the number of microglia is increased in the brains of postmortem in patients with alcohol dependence.^14^ Alcohol also affects the peripheral immune system, leading to thiamine deficiency and increases in Aβ precursors in the brain, particularly in the transgenic mouse models of AD where thiamine deficiency has been shown to exacerbate amyloid plaque pathology.^15^

Phosphodiesterase is a superfamily of enzymes that catalyzes the hydrolysis of cAMP and/or cyclic guanosine monophosphate. There are 11 families of PDE (PDE1-11) that play an important role in regulating the intracellular levels of these 2 second messengers. Phosphodiesterase-4, as one of the most important PDEs, is essential in the regulation of CNS function^16^ as it is responsible for 70%-80% of cAMP hydrolysis in neuronal cells in the brain and is a key regulator of intracellular cAMP levels. Thus, PDE4 inhibitors have a variety of central effects, including antidepressant, anxiolytic, anti-alcohol addiction, and enhanced memory and cognitive function.^17-20^ Our group has demonstrated for the first time that PDE4 plays an important role in regulating drinking behavior^17^; PDE4 inhibitors, such as rolipram, reduce alcohol consumption and preference and inhibit alcohol self-administration in alcohol-preferring animals by increasing cAMP contents in the brain.^21,22^ The main intracellular signaling pathway is that the increase in cAMP in the brain activates protein kinase A (PKA), which then phosphorylates cAMP response element-binding protein (CREB) to form phosphorylated CREB (pCREB). The latter increases brain-derived neurotrophic factor (BDNF). This signaling pathway is closely related to alcohol drinking behavior; activation of cAMP signaling inhibits alcohol drinking, and vice versa.^23^ Meanwhile, PDE4 plays an important role in the mediation of AD.^18^ Inhibition of PDE4 in the brain improves learning and memory ability by activating the cAMP-PKA-CREB signaling pathway and protecting against the decline of memory ability in AD. The cAMP signaling is considered to be a common pathway that regulates alcohol dependence and AD. Therefore, it was reasonable to believe that PDE4 could be used as a target for the treatment of AlD; this has not been investigated. In addition, to date, none of the PDE4 inhibitors has been clinically used to treat diseases associated with their central effects. One important reason is that the role of PDE4 isoforms is unclear due to the lack of highly selective PDE4 isoform inhibitors. In addition, the side effects such as emesis and vomiting induced by general PDE4 inhibition have also hindered the development of new drugs with PDE4 inhibitory abilities.

Therefore, the development of highly selective PDE4 subtype inhibitors with high potency and low toxicity has been an important research direction in this field. PDE4 is the most complex PDE family, consisting of 4 isoforms (PDE4A-D), which are expressed by 4 different genes. While PDE4C is mainly distributed in peripheral tissues, the other 3 PDE4 isoforms are highly expressed in the brain with brain region specificity, suggesting that these isoforms may have different central functions. Phosphodiesterase-4Dis highly distributed in the medulla oblongata vomiting center,^24^ which accounts for the side effects such as emesis and vomiting caused by PDE4D inhibition.^25^ PDE4B is mainly distributed in the cerebral cortex, striatum, amygdala, and hippocampus^26^; it is involved in alcohol addiction and dementia.^27,28^ In addition, studies have shown that PDE4B is highly expressed in microglia^29^; PDE4B inhibitors have potent anti-inflammatory effects, and PDE4 plays a major role in alcohol-induced neuroinflammation.^30^ A33, a relatively selective PDE4B inhibitor, has been used for studying the targeting role of PDE4B in various diseases.^31^ A33 binds to a single amino acid in the C-terminus of PDE4B to promote the closing of this domain over the active site of the enzyme, thereby preventing access to cAMP and its hydrolysis.^31,32^ This mechanism of inhibitory action confers a 100-fold greater selectivity of A33 for PDE4B (IC50 = 15 nM) over PDE4D (IC50 = 1.7 µM) and other PDEs (IC50 > 10 µM), with a half-life of approximately 4 hours in mouse brains.^33,34^ Therefore, we investigated the effects of A33 on alcohol consumption, learning and memory, and histopathological features in the mouse model of AlD. In addition, we also examined its effects on various proteins associated with neuroinflammation and cAMP signaling molecules to identify the underlying mechanisms of the actions. The results demonstrated that PDE4, in particular PDE4B, is importantly involved in alcohol drinking and cognition; PDE4B inhibitors may be a novel class of drugs for the treatment of AlD.

## MATERIALS AND METHODS

### Cell Cultures

Wild-type (WT)-7 cells, which are Neuro-2a cells stably transfected with APP/PS1 genes, were purchased from the Haixing Biotechnology Company. Cells were cultured in the Dulbecco’s Modified Eagle’s medium/F12 medium (Gibco) supplemented with 10% (v/v) heat-killed fetal bovine serum and a penicillin-streptomycin mixture (1:100; Gibco), The cells were incubated at 37 °C with 95% air and 5% CO_2_ in a humid atmosphere.

### Animals

Triple transgenic mice (3xTg-AD) harboring 3 mutant genes PS1_M146V_, APP_swe_, and tau_P301L_ were purchased from the Jackson Laboratory and used in the experiments as previously described.^35,36^ Given that the mice are homozygous for the mutations in the PS1, APP, and tau genes, we maintained the colony by breeding homozygous 3xTg-AD mice with each other.^37^ Non-transgenic, aged-matched, WT mice (C57BL6/129SvJ) served as the control. Each group consisted of 10 mice, half male and half female. Each mouse was housed individually in a clean polypropylene cage and maintained on a 12-hour-light/dark cycle (lights on from 8:00 AM to 8:00 PM) at 25 °C ± 2 °C and 40%-50% relative humidity. Food and water were freely available. The experimental procedures were carried out according to the requirements of the Laboratory Animals’ Ethics Committee of Qingdao University.

### Behavioral Tests

#### The 2-Bottle Choice Test

Three-month-old 3xTg-AD and WT mice of the same genetic background were subjected to a 19-week 2-bottle choice voluntary alcohol drinking. Briefly, in order to generate the mouse model of AlD, mice were given continuous 24-hour access to 2 100-mL bottles containing sweetened alcohol (25%, w/v + saccharin 0.1%, w/v) or water for 19 weeks until sacrificed, and drug treatment was started at week 17. The parallel control group was given 2 consecutive bottles containing saccharin (0.1% w/v) or water. Measurements of fluid consumption and the position of the bottle, left or right, were alternated every 24 hours to avoid side bias.

**Figure 1.**
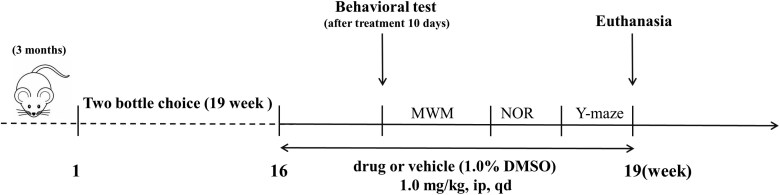
**Experimental design.** Three-month-old mice were subjected to 19 weeks of 2-bottle choice alcohol (25%, v/v) drinking and 21 days of drug treatment starting at week 16. From days 11 to 20 after the beginning of drug treatment, behavioral tests were performed 1 hour after administration in the following order: Morris water maze (MWM), novel object recognition (NOR), and Y-maze tests were used. On day 21, all mice were euthanized, and the brains were collected for histology and biochemistry analysis.

Starting on the second day after the 10th injection (i.p.) of rolipram or A33, behavioral tests were conducted in the following order: the Morris water maze (MWM), novel object recognition (NOR), and Y-maze tests (Figure 1). Tests were conducted 1 hour after rolipram treatment to minimize its potential residual sedation. The mouse was returned to its home cage after each test.

#### The MWM Test

This was performed as described previously.^38^ In brief, a circular pool with a diameter of 120 cm and a height of 50 cm was filled with opaque water 30 cm deep at 23–25 °C. The pool was divided into 4 equal quadrants. The cylindrical escape platform was placed in the center of the fourth quadrant and its top was 1 cm below the water surface. Mice were trained by successively placing them in the water at a location equidistant from the target platform in each quadrant for 5 days. In the place navigation test, each mouse was trained for 4 trials a day for 5 consecutive days to locate the submerged platform, and the escape latency was recorded as the arithmetic mean of the 4 trials. In each training session, mice were placed into the center of each of the 4 quadrants facing the wall and allowed to swim freely to locate the submerged platform; the mice were allowed to stay on the platform for 30 seconds. If the mouse failed to find the platform within 60 seconds, it was manually guided and allowed to remain on the platform for 30 seconds. During the spatial exploration test on day 6, the platform was removed, and the mouse was placed into the water on the opposite side of the previous platform quadrant; then the mouse was allowed to swim for 60 seconds. The time spent in the target quadrant and the number of crossings into the original platform area were recorded using the Topscan Package (Clever Sys). The latency to the platform in MWM data was assessed using repeated-measures 2-way ANOVA. Details are provided in the Supplementary Material (Figure S1)

#### The NOR Test

The NOR, which assesses cognitive ability based on rodents’ tendency to explore new objects, was performed using a white square open-field box (40 × 40 × 40 cm) for 3 consecutive days. On day 1, mice were acclimated to the empty testing arena for 5 minutes. On day 2, mice were placed in the arena and allowed to explore 2 identical objects (Cuboid Lego bricks, 5 × 5 × 7 cm) freely for 5 minute. On day 3, one of the familiar objects was replaced with a novel object (Cylinder Lego blocks, 5 × 7 cm), and mice were allowed to explore the arena for 5 minutes. After each test, each object was wiped with the 75% alcohol solution to remove potential odor cues left by the previously tested mouse so as not to interfere with the exploration of the testing mice. Recognition is defined as actively touching or facing (within 2 cm) the object. The recognition index was calculated as the percentage of time spent on the novel object over the total time for exploring both novel and old objects. Professional behavioral software was used for analysis (XinRuan).

#### The Y-Maze Test

The Y-maze test is used to assess the spatial working memory of rodents based on their innate curiosity to explore previously unvisited areas. The Y-shaped maze (30 cm long, 8 cm wide, 15 cm high) was equipped with 3 white arms at 120° angles from one another. One of the arms was set up as the starting arm zone A; one of the remaining 2 arms was blocked, called the novelty arm zone B, and the other arm was the familiar arm zone C. The test was divided into 2 phases with a 2-minute interval. In the first learning phase, the mouse was placed at the end of the arm zone A with its head toward the central area of the maze, and the mouse was allowed to freely explore the starting arm, the familiar arm, and the central area for 5 minutes. No tracking and recording were performed during this stage. After 5 minutes of exploration, mice were removed and 2 minutes later, the second (testing) phase was started. The blocked novelty arm was opened, and the mouse was placed again from the end of the starting arm in the same way. The mouse was allowed to freely explore the 3 arms for 5 minutes, and its movement was tracked and recorded during this period. After each test, each arm was wiped with the 75% alcohol solution to remove potential odor cues left by the previously tested mouse so as not to interfere with the exploration of the testing mouse. The camera and SMART V3.0 video tracking system were used to record the time of the mouse arm movement in the Y-maze during the second phase of testing.

### Drugs and Treatments

Rolipram and A33 were purchased from TargetMol. They were dissolved in saline containing 1% dimethyl sulfoxide (DMSO). All mice (*n* = 10 per group) were treated with the drug after 16 weeks of 2-bottle choice before being divided into the following groups: WT drinking water (WT + W, 1% DMSO), WT drinking alcohol (WT + A, 1% DMSO), 3xTg-AD drinking water (3xTg-AD + W, 1% DMSO), 3xTg-AD drinking alcohol (3xTg-AD + A, 1% DMSO), 3xTg-AD drinking alcohol with rolipram (3xTg-AD + A + R; rolipram, 1 mg/kg), and 3xTg-AD drinking alcohol with A33 (3xTg-AD + A + A33_;_ A33, 1 mg/kg). All animals received once daily (8 AM) i.p. injections of drug or vehicle in a total volume of 10 mL/kg body weight (Figure 1).

### Preparation of Brain Tissues

After behavioral testing, all mice were anesthetized with isoflurane and euthanized (4 PM). The brains of 4 randomly selected animals from each group were fixed overnight in 4% paraformaldehyde, embedded in paraffin with a microslicer (Leica), and mounted on glass slides for histopathology and immunofluorescence staining (see below). The rest of the brain was carefully dissected frozen on ice and stored at −80 °C until analysis was completed.

### Western Blotting

Brain tissues were homogenized in r**adio**i**mmunoprecipitation** a**ssay** (RIPA) **Lysis Buffer**‌ containing protease and phosphatase inhibitors and centrifuged at 13200 x g at 4 °C for 15 minutes. The supernatant was transferred to a clean tube and assayed for total protein using a bicinchoninic acid (BCA) kit, then equal amounts of protein were separated on sodium dodecyl sulfate-polyacrylamide gels and transferred to polyvinylidene difluoride membranes. Membranes were blocked with 5% nonfat dry milk in tris-buffered saline (TBS) containing 0.1% Tween-20 for 1 hour at room temperature, followed by overnight incubation at 4 °C with primary antibodies. The membranes were then washed with TBS containing 0.1% Tween-20, incubated for 1 hour at room temperature with secondary antibodies, and washed again. Bands were visualized with an enhanced chemiluminescence detection kit and scanned using an AI-680 System (GE). The amount of each protein was normalized for the amount of the corresponding β-actin detected in the sample. Details of antibody dilution are provided in the Supplementary Material (Figure S2)

### Enzyme-Linked Immunosorbent Assay

Tissues from the hippocampus were homogenized in ice-cold phosphate buffered saline (PBS) and centrifuged at 2000 rpm for 20 minutes at 4 °C; the supernatant was then assayed for IL-1β, IL-6, TNF-α, using enzyme-linked immunosorbent assay (ELISA) kits following the manufacturer’s instructions (Jiangsu Meibiao Biotechnology). Absorbance at 450 nm was measured using a multifunctional microplate reader.

### Immunofluorescence Staining

Tissue slices were dried in an oven at 60 °C, dewaxed, rehydrated, subjected to antigen retrieval, blocked with 3% bovine serum albumin (BSA) for 30 minutes at room temperature, and incubated overnight at 4 °C with anti-glial fibrillary acidic protein (GFAP) (GB11096,1:200, Servicebio) and anti-Iba1 (GB11105,1:200, Servicebio) antibodies, washed for 3 times with PBS for 15 minutes, incubated with secondary antibodies for 1 hour in darkness at room temperature, stained with 4′,6-diamidino-2-phenylindole for 10 minutes, and then incubated with the spontaneous fluorescence quenching reagent for 5 minutes. The sections were washed with PBS again and mounted on microscope slides with the anti-fade mounting medium. Images were captured using a slice scanner (Pannoramic MIDI).

### The Enzyme Activity Test

This was performed as described previously.^39^ In brief, hippocampal supernatants were used as PDE4 enzyme, cAMP was used as the substrate, and another group of supernatants was added with rolipram to inhibit PDE4 activity. Enzymatic activity was expressed as the difference between the remaining cAMP peaks in high performance liquid chromatography (HPLC) for these 2 groups.

### Immunochemistry

The sections were deparaffinized, rehydrated, subjected to antigen retrieval, and then rinsed in distilled water. The sections were blocked with 3% BSA (G5001, Servicebio) for 30 minutes, and then incubated with anti-Aβ-antibodies (25524-1-AP, 1:200, Proteintech) at 4 °C overnight. The secondary antibodies (horseradish peroxidase [HRP] labeled) of the corresponding species were then added to cover the tissues, and incubated at room temperature for 60 minutes. The slides were then placed in PBS (PH7.4) for decolorization. After the sections were dried a little, freshly prepared diaminobenzidine (DAB) chromogenic solution was dripped in the circle. The color development time was controlled under the microscope. The positive color was brown. Rinse the sections with double distilled water to stop the color display. Hematoxylin was counter-stained for about 3 minutes, washed with double distilled water, differentiated with hematoxylin differentiation solution for a few seconds, rinsed again with double distilled water, and as the hematoxylin blue solution turned blue, rinsed with running water. Finally, the slices were placed in the following conditions for dehydration and transparent: 75% and 85% alcohol, anhydrous ethanol I and II, and xylene I, each for 5 minutes. Sections were removed from toluene and allowed to dry before the slides were covered with neutral gum.

### Statistical Analysis

Data shown are expressed as means ± SEM. All data were analyzed using GraphPad Prism version 8 (GraphPad Software). The normality of data was verified using the Shapiro-Wilk test, and their homogeneity of variance was verified using the Brown-Forsythe test. Differences among multiple groups were assessed for significance using 2-way (genotype × treatment) ANOVA followed by post hoc Tukey tests. Due to the large number of experimental groups, each group was compared with the 3xTg-AD + A group: * *P* < .05, ** *P* < .01, *** *P* < .001, **** *P* < .0001. In order to make the experimental data cleaner, we did not label the groups without significant changes. We added relevant details to the figure legends. The latency to the platform in MWM data was assessed using repeated-measures 2-way ANOVA. Differences with *P* < .05 were considered statistically significant.

## RESULTS

### A33 Alleviated Alcohol-Exacerbated Memory Deficits in 3xTg-AD Mice

**Figure 2.**
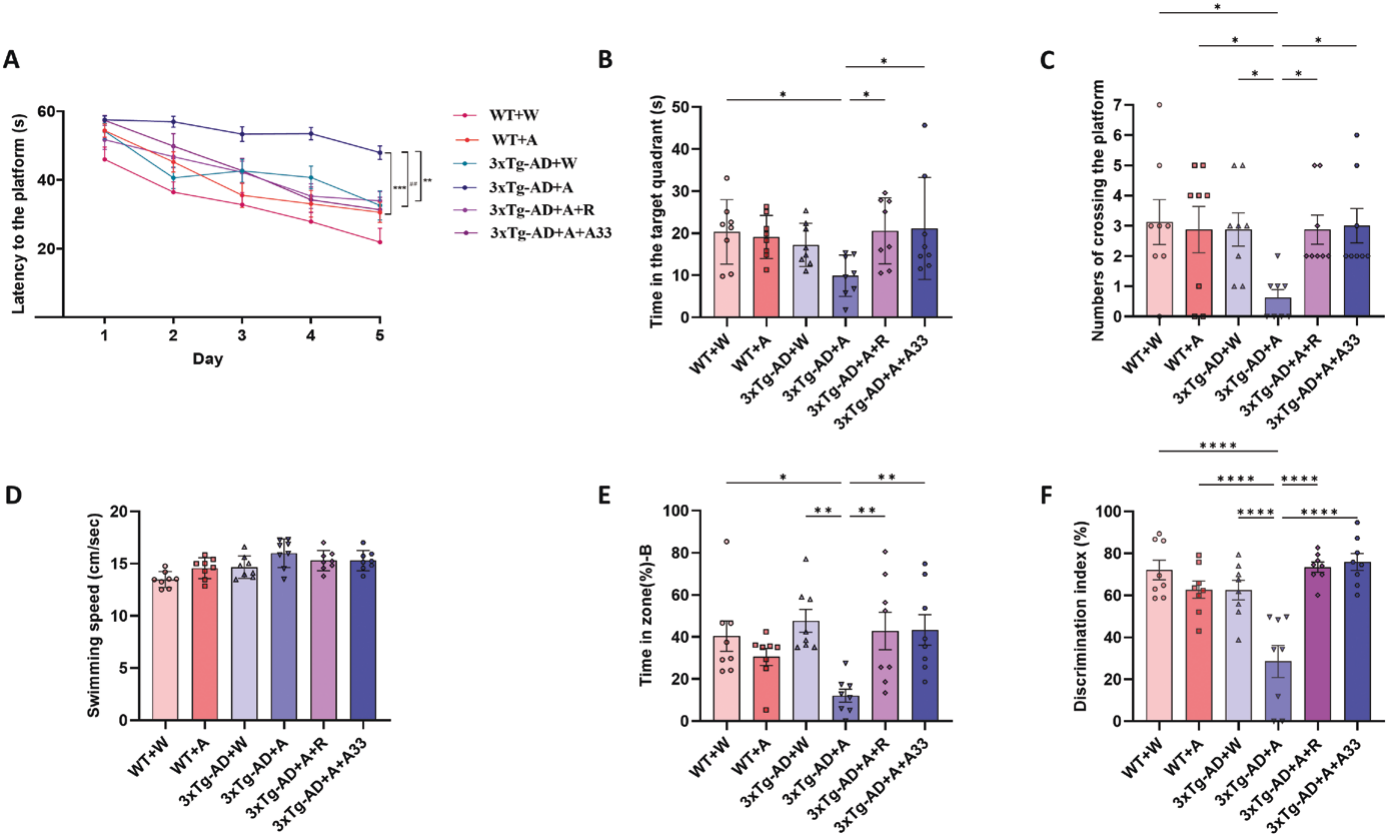
**Effects of the PDE4B inhibitor A33 on behaviors in the Morris water maze (MWM), Y-maze, and novel object recognition (NOR) tests in alcoholic dementia mice.** (A) Escape latency, (B) time spent in the target quadrant, (C) numbers of crossing the platform, and (D) swimming speed in the MWM test. (E) The proportion of time spent in novelty zone B in the Y-maze. (F) The recognition index in the NOR test. Bars represent means ± standard error of the means (SEM). *P < .05, **P < .01, ****P < .0001 compared with the 3xTg-AD + A group at the same time point; *n* = 8 per group. PDE4B, Phosphodiesterase-4B.

Behavioral experiments were conducted to investigate the impact of alcohol on learning and memory and the potential improvement of A33 in AlD mice. On day 5 during the acquisition training, compared to the 3xTg-AD + W group, 3xTg-AD + A mice exhibited significantly impaired learning and cognition, as evidenced by a longer time required to reach the escape platform; treatment with rolipram or A33 resulted in a significant reduction in the time finding the platform, as indicated in both 3xTg-AD + A-R and 3xTg-AD + A-A33 groups (Figure 2A). After removing the platform on day 6, there was a tendency for decreased time spent in the target quadrant and fewer platform crossings in the 3xTg-AD + A group, relative to the 3xTg-AD + W control. However, these effects were significantly increased after A33 treatment (Figure 2B and C). No differences were found in swimming speed between groups (Figure 2D). In the Y-maze test, mice in the 3xTg-AD + A group spent significantly less time exploring novelty arm B compared to the 3xTg-AD + W controls (Figure 2E), indicating impaired spatial working memory. Similarly, in the NOR test, mice in the 3xTg-AD + A group spent less time exploring novel objects than their counterparts in the 3xTg-AD + W group, suggesting defective episodic memory (Figure 2F). The selective PDE4B inhibitor A33 effectively reversed both conditions with comparable efficacy to general PDE4 inhibitors (Figure 2E and F).

### Alcohol Upregulated the Expression of Aβ, APP, and PS1 in WT-7 Cells and 3xTg-AD Mice, While A33 Reversed the Upregulated Expression of Aβ

**Figure 3.**
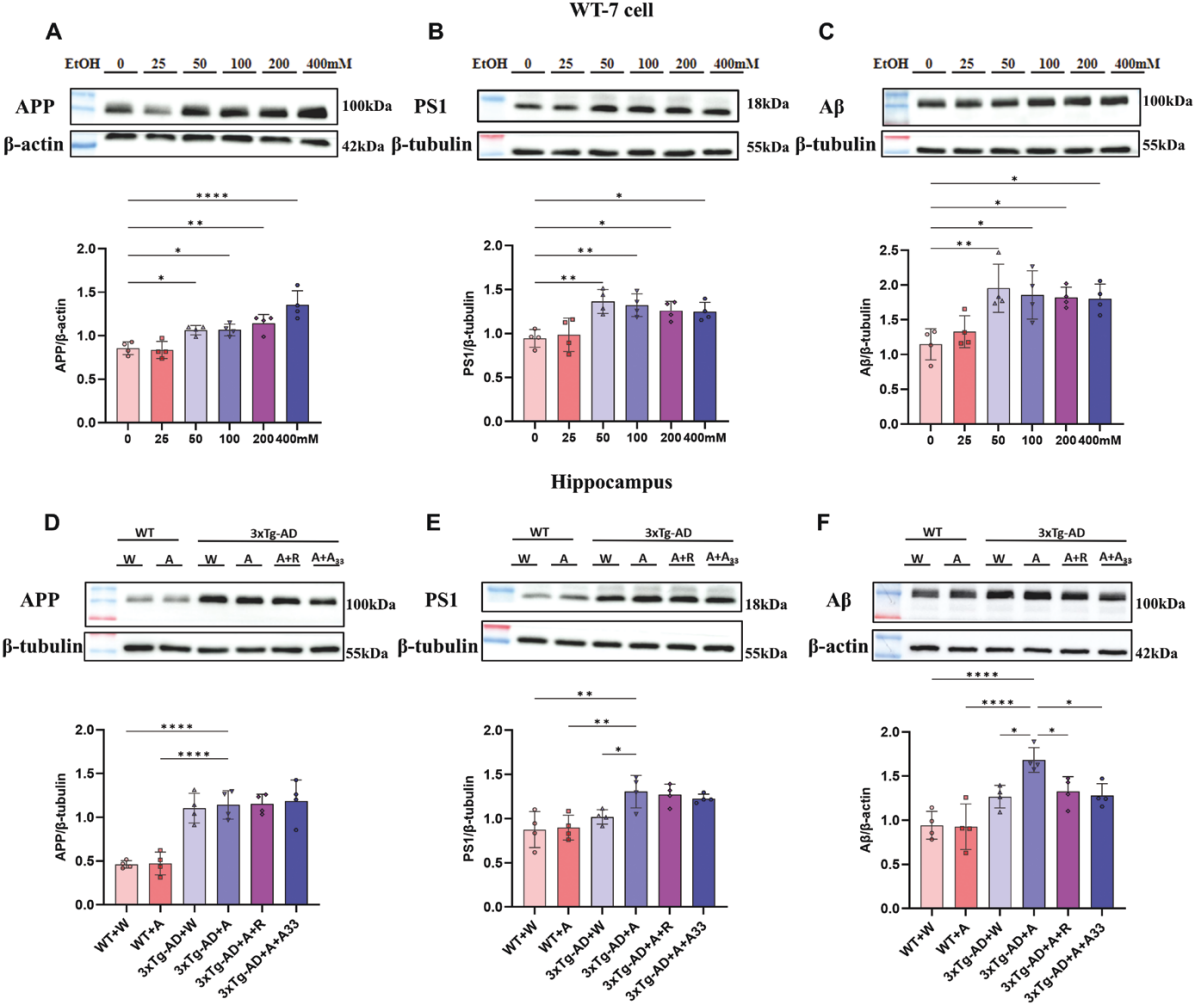
**The expression of APP, PS1, and Aβ in WT-7 cells and AlD mice.** (A) APP, (B) PS1, and (C) Aβ in WT-7 cells with different alcohol concentrations. Bars represent means ± standard errors (SEM). *P < .05, **P < .01, ****P < .0001 compared with the WT cells at the same time point; *n* = 4. (D) APP, (E) PS1, and (F) Aβ in the mouse hippocampus. Bars represent means ± standard errors (SEM). *P < .05, **P < .01, ****P < .0001 compared with the 3xTg-AD + A group at the same time point; *n* = 4. AID, alcoholic dementia; WT, wild type.

To investigate the effects of alcohol on the progression of AD, we first examined the effects of alcohol on AD-related proteins such as APP and PS1 and AD’s hallmark product Aβ in WT-7 cells. The cells were exposed to varying concentrations of ethanol to examine the effects of ethanol on the expression of APP, PS1, and Aβ proteins. At the concentration of 50 mM, alcohol significantly increased the expression of these proteins (Figure 3A-C). Within a certain range, increasing ethanol concentrations led to elevated APP expression. Consistent with this, compared to the WT control, 3xTg-AD mice after alcohol consumption (ie, 3xTg-AD + A) showed significantly higher expression of both APP and PS1 proteins in the hippocampus. Additionally, the 3xTg-AD + A group exhibited significantly higher levels of PS1 compared to 3xTg-AD + W (Figure 3D and E). Similarly, the 3xTg-AD + A group demonstrated significantly higher levels of Aβ than the 3xTg-AD + W group; this was substantially reduced following treatment with rolipram or A33 (Figure 3F).

### A33 Prevented the Deposition of Aβ Plaques in AlD Mice

**Figure 4.**
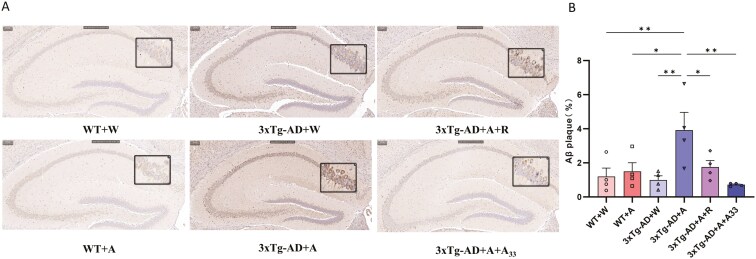
**The immunohistochemistry of Aβ plaques in the hippocampus of AlD mice.** (A) Immunohistochemical results of Aβ plaques in the hippocampus. (B) Quantitative statistics of Aβ plaques in hippocampal tissues of different groups. Bars represent means ± SEM. *P < .05, **P < .01 compared with the 3xTg-AD + A group at the same time point; *n* = 4. AID, alcoholic dementia.

Immunohistochemical detection of Aβ plaque depositions in the brain revealed that the hippocampal Aβ plaques in the 3xTg-AD + A group were significantly increased after alcohol consumption; this was reversed by treatment with rolipram or A33 (Figure 4A and B).

### A33 Reduced Alcohol Consumption and Alcohol Preference in Mice

**Figure 5.**
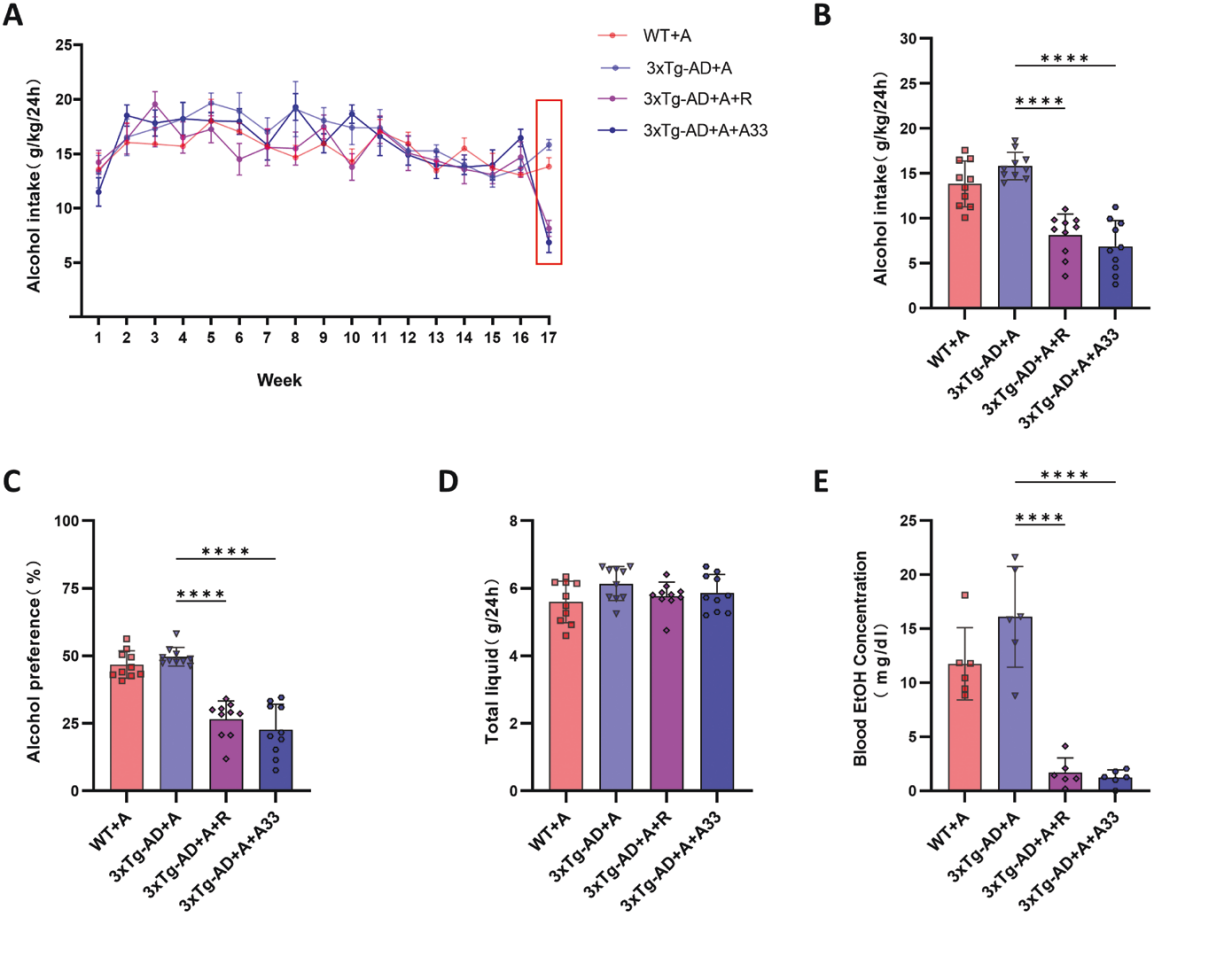
**Effects of A33 on alcohol intake and preference in WT and 3xTg-AD mice exposed to alcohol using 2-bottle choice.** (A) Alcohol intake during the first 17 weeks. (B) Alcohol intake at week 17. (C) Alcohol preference at week 17. (D) The total liquid intake at week 17. (E) Blood alcohol concentration after sacrifice. Bars represent means ± SEM. ****P < .0001 compared with the 3xTg-AD + A group at the same time point; *n* = 10 except for (E) (*n* = 6). WT, wild type.

Three-month-old 3xTg-AD and WT mice of the same genetic background were subjected to a 19-week 2-bottle choice experiment, and drug treatment was started at week 17. There were no significant differences in alcohol intake between groups of mice during the first 16 weeks (Figure 5A). At week 17, alcohol intake and alcohol preference of mice in 3xTg-AD + A + R and 3xTg-AD + A + A33 groups were significantly lower than those in the 3xTg-AD + A group (Figure 5B and C). In contrast, the total daily fluid intake was not changed between groups (Figure 5D). After the mice were euthanized, serum was collected for measurement of blood alcohol concentration, which was significantly lower in the 3xTg-AD + A + R and 3xTg-AD + A + A33 groups compared to the 3xTg-AD + A group (Figure 5E).

### Effect of A33 on PDE4 Enzymatic Activity and the Downstream Substrate cAMP in the Hippocampus

**Figure 6.**
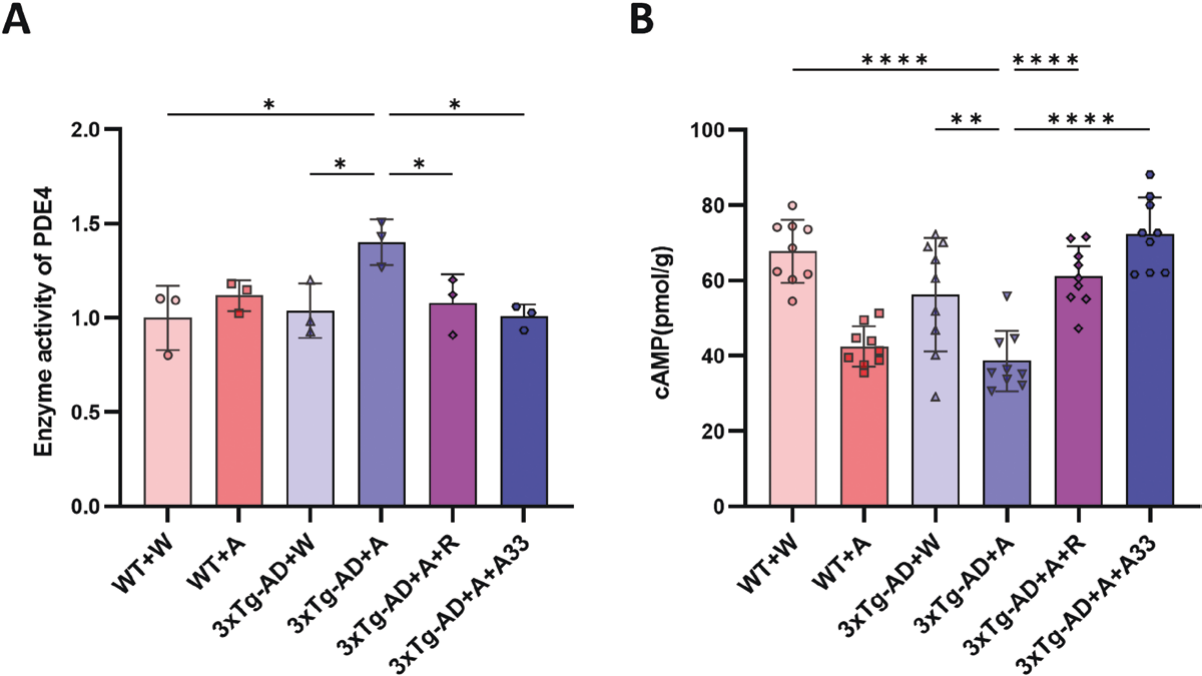
**Effects of A33 on PDE4 activity and cAMP levels in the hippocampus of WT and 3xTg-AD mice exposed to alcohol.** (A) PDE4 activity by HPLC (*n* = 3). (B) The cAMP content by ELISA (*n* = 9). Bars represent means ± SEM; *P < .05, **P < .01, ****P < .0001 compared with the 3xTg-AD + A group at the same time point. PDE4, phosphodiesterase-4.

The PDE4 enzyme activity was quantified by measuring the difference in cAMP peak values using HPLC. Upon alcohol consumption, PDE4 activity was significantly increased in 3xTg-AD mice; this was decreased by treatment with rolipram or A33, as shown in the 3xTg-AD + A + R and 3xTg-AD + A + A33 groups, respectively, relative to the 3xTg-AD + A group (Figure 6A). Consistent with this, the level of cAMP was significantly lower in the 3xTg-AD + A group compared to 3xTg-AD + W and, similarly, there were significant increases in cAMP levels in both the 3xTg-AD + A + R and 3xTg-AD + A + A33 groups (Figure 6B).

### Effects of A33 on Expression of PDE4 Subtypes and Signaling Components in the Hippocampus

**Figure 7.**
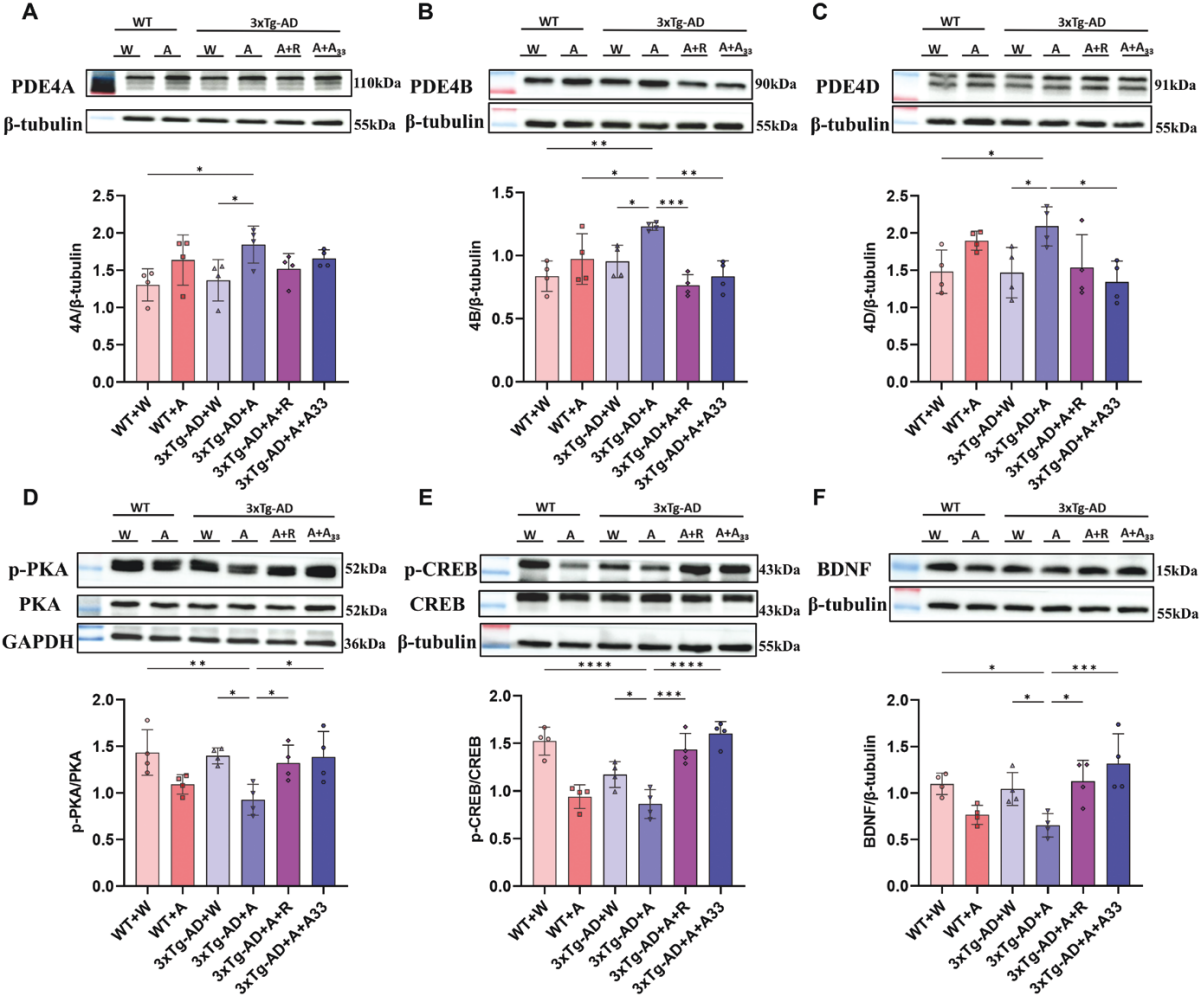
**Effects of A33 on the expression of PDE4 subtypes and the downstream proteins.** (A) PDE4A, (B) PDE4B, (C) PDE4D, (D) p-PKA, (E) p-CREB, and (F) BDNF in the hippocampus of WT and 3xTg-AD mice exposed to alcohol. Bars represent means ± SEM. *P < .05, **P < .01, ***P < .001, ****P < .0001 compared with the 3xTg-AD + A group at the same time point (*n* = 4). BDNF, brain-derived neurotrophic factor; PDE4, phosphodiesterase-4; p-CREB, phosphorylated cAMP response element-binding protein.

After the 19-week alcohol drinking, the expression of PDE4A, 4B, and 4D in the 3xTg-AD + A group was significantly higher relative to the 3xTg-AD + W group. Additionally, the expression of PDE4B in 3xTg-AD + A was significantly higher compared to WT + W, WT + A, and 3xTg-AD + W. Following treatment with rolipram or A33, the levels of PDE4B in 3xTg-AD + A remained significantly higher relative to 3xTg-AD + A + R and 3xTg-AD + A + A33 (Figure 7A-C). Furthermore, compared to 3xTg-AD + W, the 3xTg-AD + A group displayed significant decreases in the expression of p-PKA and p-CREB in the hippocampus. However, there were no differences in total PKA and CREB (Figure 7D-F). Moreover, the expression of p-PKA and p-CREB in the hippocampus of WT-A mice was also decreased compared to that of WT-W mice. Additionally, the expression of BDNF in the hippocampus of 3xTg-AD + A mice was significantly decreased compared to that of 3xTg-AD + W mice; this was reversed by A33 or rolipram treatment (Figure 7D-F).

### Effects of A33 on Microglial Activation and Neuroinflammation in the Hippocampus of AlD Mice

**Figure 8.**
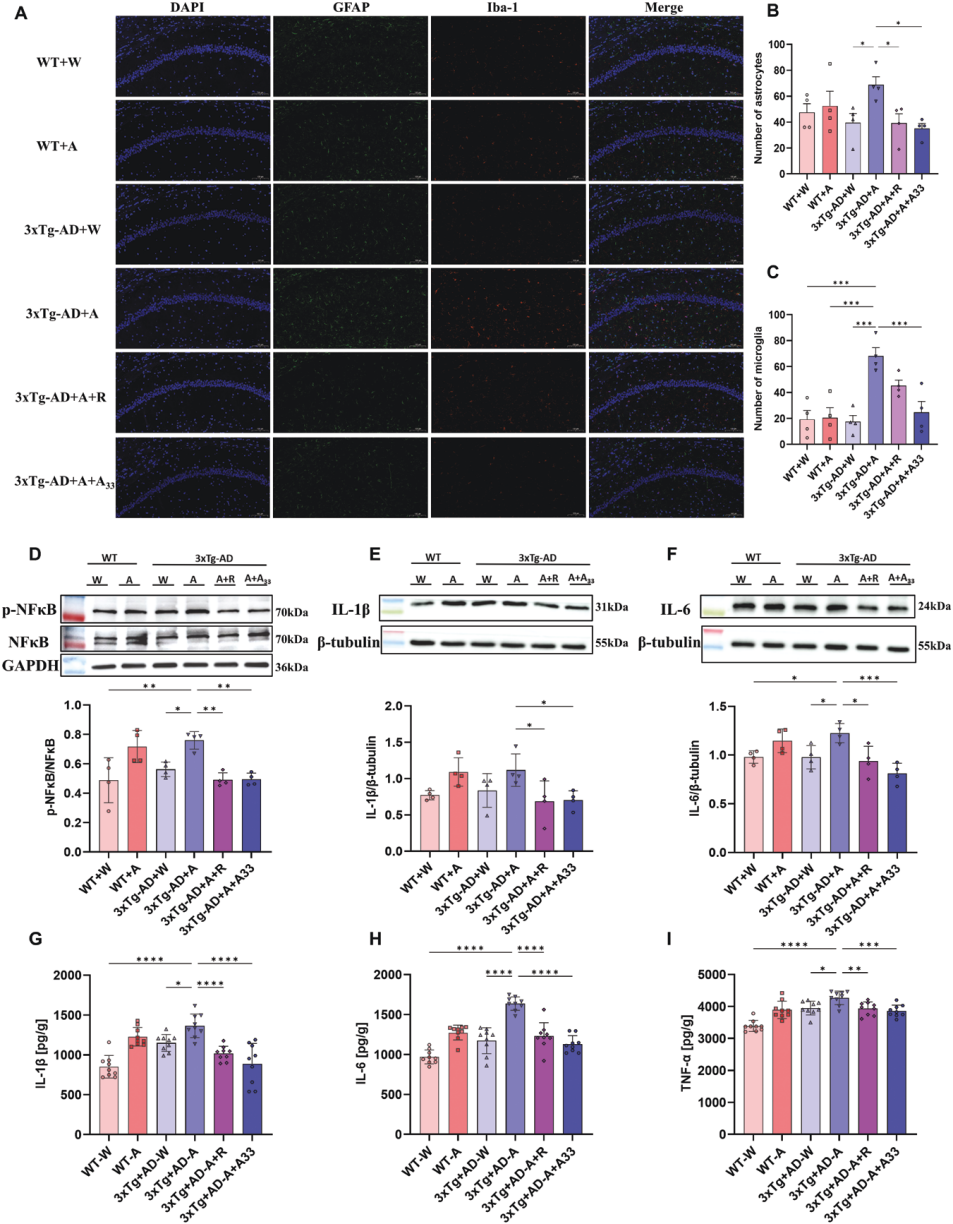
**A33 attenuated the inflammatory responses in the hippocampus of AlD mice using immunofluorescence (A-C), Western blotting (D-F), and ELISA (G-I).** (A) Immunostaining of hippocampal slices with anti-GAFP (green), anti-Iba1(red), and DAPI (blue). (B, C) Quantification of astrocytes and microglia. (D) Immunoblotting of p-NF-κB, (E) IL-1β,and (F) IL-6 in the hippocampus. (G) ELISA analysis of IL-1β, (H) IL-6, and (I) TNF-α in the hippocampus. Bars represent means ± SEM. *P < .05, **P < .01, ***P < .001, ****P < .0001 compared with the 3xTg-AD + A group at the same time point; *n* = 4 for A-F or 9 for G-I. AID, alcoholic dementia; DAPI, 4',6-diamidino-2-phenylindole.

The group of 3xTg-AD + A mice administered with alcohol exhibited a higher number of GFAP-positive cells (ie, activated astrocytes) and Iba1-positive cells (ie, activated microglia) in the hippocampal CA1 region than 3xTg-AD + W mice; the increase in GFAP-positive cells was reversed by rolipram treatment. Comparably, there was only a downward trend observed for Iba1-positive cells after rolipram treatment, which was subsequently reversed by A33 administration (Figure 8A-C). Immunoblotting analysis revealed that p-NF-κB, IL-1β, and IL-6 levels in the hippocampus of WT + A and 3xTg-AD + A mice were also significantly or slightly increased after alcohol administration, and this trend was reversed after rolipram or A33 administration (Figure 8D-F). Consistently, ELISA analysis revealed that IL-1β, IL-6, and TNF-α levels in the hippocampus of 3xTg-AD + A mice were also significantly increased after alcohol administration, which was reversed after rolipram or A33 administration (Figure 8G-I). However, there was no significant change in serum IL-1β, IL-6, and TNF-α levels (Figure S3).

## DISCUSSION

In the present study, we demonstrated for the first time that the PDE4B inhibitor A33 prevented cognitive impairment and Aβ deposition in an animal model of AlD, primarily by alleviating neuroinflammatory reactions in the hippocampus via the activation of the PDE4B/cAMP/PKA signaling pathway. A33-treated AlD mice showed better cognitive abilities than AlD mice treated with vehicle. In addition, administration (i.p.) of A33 in AlD mice attenuated neuropathological damage in the hippocampus. More specifically, A33 reduced Aβ deposition and Aβ expression in AlD mice, along with increased pCREB and BDNF expression, which may result from the activation of cAMP signaling. Moreover, A33 significantly decreased the expression levels of inflammatory cytokines (eg, IL-1β, IL-6, and TNF-α) and NF-κB, as well as the activation of microglia in the hippocampus of AlD mice, leading to attenuating inflammatory responses. In addition, A33 administrated for 3 weeks reduced alcohol consumption in AlD mice using 2-bottle choice alcohol drinking. These results suggest that A33 ameliorates cognitive impairment of AlD by reduction of Aβ burden, inhibition of microglial cells, and recovery of neurogenesis via PDE4B-mediated cAMP/PKA signaling (Figure S4).

We have previously demonstrated that the PDE4 inhibitor rolipram reverses memory deficits in APP/PS1/tau transgenic mice by participating in neuroinflammation and apoptosis through cAMP signaling.^40^ Recent studies have also found that alcohol exacerbates neural and behavioral pathology in 3xTg-AD mice.^4^ Given the dual effects of AD and alcohol on brain regions, particularly the hippocampus which is closely associated with learning, memory, and alcohol addiction, it was of interest to investigate the potential changes in different subtypes of PDE4 in the hippocampus of AlD mice and identify the most effective therapeutic target for AlD.^27,28^ This is particularly crucial for avoiding adverse reactions induced by general inhibition of PDE4.

Our data revealed that the expression of PDE4A, PDE4B, and PDE4D was increased to different extents in response to alcohol exposure. Using gene knockout (KO), we have demonstrated the involvement of PDE4A in the regulation of cognition and emotion.^41^ Notably, PDE4A KO mice display enhanced cognitive ability.^42^ Due to the limited distribution in addiction-related brain regions such as the striatum and bed nucleus of stria terminalis, it is generally believed that PDE4A has a minimal impact on alcohol drinking behavior. In contrast, PDE4B is highly expressed in the striatum, amygdala, and the bed nucleus of the stria terminalis,^24^ indicating that PDE4B may be a key player in alcohol dependence and abuse. It has been shown that selective PDE4B inhibitors reduce alcohol consumption in mice.^43^ This is supported by the findings from recent studies. A33, a relatively selective inhibitor of PDE4B, reduces alcohol drinking behavior in both male and female mice without affecting their locomotor activity or causing sedative side effects.^43^ Consistent with this, C57BL/6 mice subjected to alcohol diet for 1 week exhibit significant increases in the expression of PDE4B in the brain along with decreased cAMP levels. This is accompanied by glial cell activation and the release of inflammatory factors.^28^ Inhibitors targeting PDE4B produce potent anti-inflammatory effects. A33 increases cAMP levels while reducing inflammatory factors in mouse brains; it reverses cognitive impairment. Although PDE4D is implicated in memory regulation,^44,45^ its prominent distribution in the chemoreceptor trigger zone of the medulla^46^ and the evident emetic-like behavior observed in mice lacking PDE4D present significant challenges for the development of cognitive-enhancing drugs targeting this isoform. Consequently, our research focus has been directed toward investigating PDE4B.

The in vitro experiments demonstrated a positive correlation between different concentrations of alcohol and the levels of AD-related proteins, including APP, PS1, and Aβ, in stable transfected WT-7 cells. This is consistent with the in vivo experiments. Alcohol exposure significantly increased the expression of PS1 and Aβ proteins in AD mice, which can exacerbate the occurrence of AD. However, compared to the in vitro observations, no significant change was observed in APP protein levels, possibly due to the elevated base levels in the brain of the AD mice. Treatment with A33 effectively reduced alcohol-induced Aβ protein expression. This study highlights that inhibition of PDE4B can effectively decrease alcohol intake and preference while providing therapeutic benefits for AD; this is associated with the upregulation of cAMP and its downstream phosphorylation of PKA/CREB. In the present study, we focused on the hippocampus as it is strongly implicated in learning, long-term memory consolidation, and retrieval processes—particularly within the hippocampal CA1 subregion.^47^ The 3xTg-AD mice at 7 months of age drinking water had no Aβ plaque deposits, likely because 3xTg-AD mice normally start to exhibit Aβ plaque deposits at 8 months of age.^37^ In contrast, after alcohol exposure, 3xTg-AD mice at the same age exhibited deposition of Aβ plaques in the brain; this effect was mitigated by treatment with A33.

As a neurotrophic factor, BDNF facilitates neuronal plasticity and survival, which is consistent with reduced levels of BDNF in patients with dementia.^48^ A33-induced increases in BDNF may rebalance the dynamics in the brain from neurodegeneration toward neural regeneration,^49^ accounting for the potential mechanism whereby A33 alleviated the behavior impairment in AlD mice. In addition, the phosphorylation levels of PKA and CREB were significantly diminished in the hippocampus of mice exposed to alcohol (3xTg-AD + A) compared to the 3xTg-AD mice to water (3xTg-AD + W). Similarly, administration of alcohol to WT (WT + A) mice resulted in notably lower phosphorylation levels of PKA and CREB in the hippocampus compared to the WT + W group, suggesting that alcohol disrupts the cAMP/PKA/CREB signaling. A33 restored the decreased phosphorylation and BDNF expression, reversing cognitive impairment in AlD mice.

Studies have demonstrated that ameliorating neuroinflammation mediated by microglia can attenuate the pathology of AD.^50^ This is consistent with our previous studies showing that the pan-PDE4 inhibitor rolipram attenuates neuroinflammation, reduces microglial activation, decreases Aβ accumulation, and enhances cognitive function in AD mice.^40^ The present study further demonstrated that selective inhibition of PDE4B by A33 ameliorated cognitive impairment in AlD mice. These therapeutic benefits are associated with the upregulation of cAMP levels and subsequent activation of the PKA/CREB signaling pathway through phosphorylation. A33 not only inhibits the hydrolysis of Aβ protein but also reduces NF-kB phosphorylation, leading to alleviating neuroinflammation and mitigating symptoms of AlD. Our study provides further evidence supporting the involvement of neuroinflammation in AlD. Given the hippocampus plays a crucial role in learning and long-term memory processes, which are significantly affected by AD pathology,^51^ we identified this brain region, specifically, hippocampal CA1, as the key player in the mediation of AlD. Alcohol exposure produced an increase in microglial cells in the CA1 region of 3xTg-AD mice, which was attenuated following treatment with A33.

## CONCLUSIONS

In summary, the results from the present study revealed that A33 mitigated the neuropathological alterations, including Aβ plaque deposition, Aβ protein expression, as well as neuroinflammation in the hippocampus, accompanied by attenuation of cognitive deficits in AlD shown by 3xTg-AD mice exposed to alcohol. Furthermore, this study demonstrates that pan-PDE4 or selective PDE4B inhibitors exert neuroprotective effects by alleviating inflammatory reactions via inhibiting PDE4, in particular PDE4B, and subsequently activating the cAMP/PKA signaling pathway in AlD. Thus, PDE4 inhibitors, especially PDE4B inhibitors are promising neuroprotective agents that can benefit for AlD-related cognitive dysfunction.

## Supplementary Material

pyaf009_suppl_Supplementary_Data

## Data Availability

The original contributions are included in the article. Further inquiries can be directed to the corresponding authors.
