# Supplementary material for: Inhibition of PDE4B ameliorates cognitive defects in the model of alcoholic dementia in 3xTg-AD mice via PDE4B/cAMP/PKA signaling
Source: Int J Neuropsychopharmacol. 2025 Feb 8;28(3):pyaf009. doi: 10.1093/ijnp/pyaf009 (PMC11923544; doi:10.1093/ijnp/pyaf009)
Supplement: pyaf009_suppl_Supplementary_Data [file pyaf009_suppl_supplementary_data.docx]

**Supplementary data**

**Sup Fig 1. Results of two-way ANOVA for escape latency in the Morris water maze test.**

A.
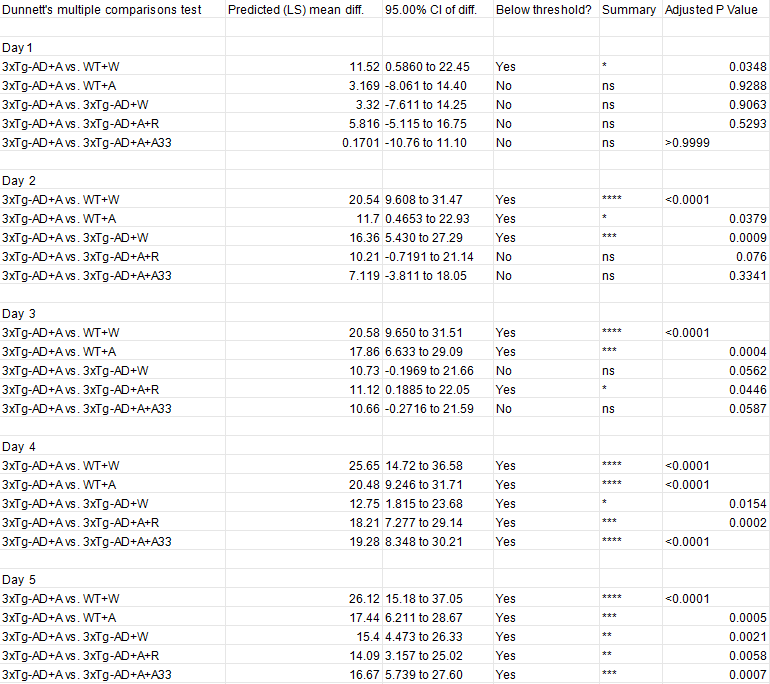


Sup Fig. 1. (A) Escape latency in the Morris water maze test. Two-way ANOVA test, bars represent mean values ± standard error of the mean (SEM). *p < 0.05, **p < 0.01, ##p < 0.01, ****p < 0.0001 compared with the 3xTg-AD+A mice at the same time point; (n = 8)

**Sup Fig 2. Details of the antibodies involved in Western Blot**

| Antibodies | Source | Identifier | Dilution |
| --- | --- | --- | --- |
| Anti-PS1 | Abcam | Cat# ab76083 | 1:1000 |
| Anti-4B | Abcam | Cat# ab170939 | 1:1000 |
| Anti-4D | Abcam | Cat# ab14613 | 1:1000 |
| Anti-PKA | Abcam | Cat# ab32514 | 1:1000 |
| Anti-p-CREB | Abcam | Cat# ab32096 | 1:1000 |
| Anti-CREB | Abcam | Cat# ab32515 | 1:1000 |
| Anti-BDNF | Abcam | Cat# ab108319 | 1:1000 |
| Anti-β-tubulin | ABclonal | Cat# AC021 | 1:5000 |
| Anti-APP | Cell Signaling Technology | Cat# 2452S | 1:1000 |
| Anti-p-PKA | Cell Signaling Technology | Cat# 5661 | 1:1000 |
| Anti-4A | proteintech | Cat# 16226-1-AP | 1:1000 |
| Anti-Aβ | proteintech | Cat# 25524-1-AP | 1:1000 |
| Anti-GAPDH | proteintech | Cat# 60004-1-Ig | 1:5000 |
| Anti-p-NF-κB | Servicebio | Cat# GB113882 | 1:1000 |
| Anti-NF-κB | Servicebio | Cat# GB11997 | 1:1000 |
| Anti-IL-1β | Servicebio | Cat# GB122059 | 1:1000 |
| Anti-IL-6 | Servicebio | Cat# GB11117 | 1:1000 |
| Anti-β-actin | Servicebio | Cat# GB15001 | 1:5000 |

**Sup Fig 3. Elisa was used to detect the related inflammatory factors in the serum of mice.**


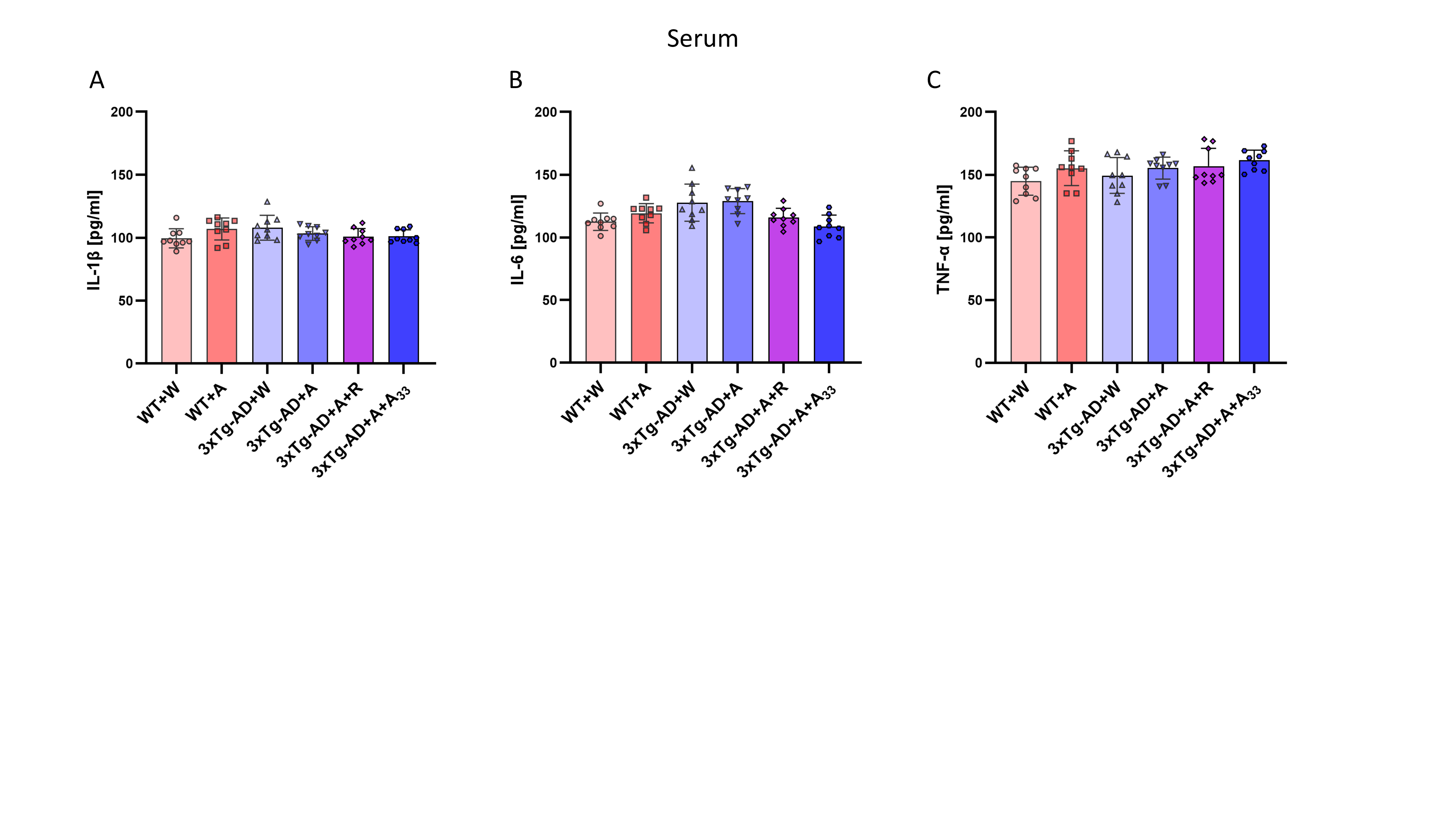


**Sup Fig. 3.** (A) IL-1β, (B) IL-6, and (C) TNF-α in the mice serum. Bars represent mean values ± standard error of the mean (SEM).*p < 0.05, compared with the 3xTg-AD+A mice at the same time point; (n = 9)

**Sup Fig 4. Schematic illustration for intracellular signaling mechanisms of the A33 actions in the treatment of alcoholic dementia.**


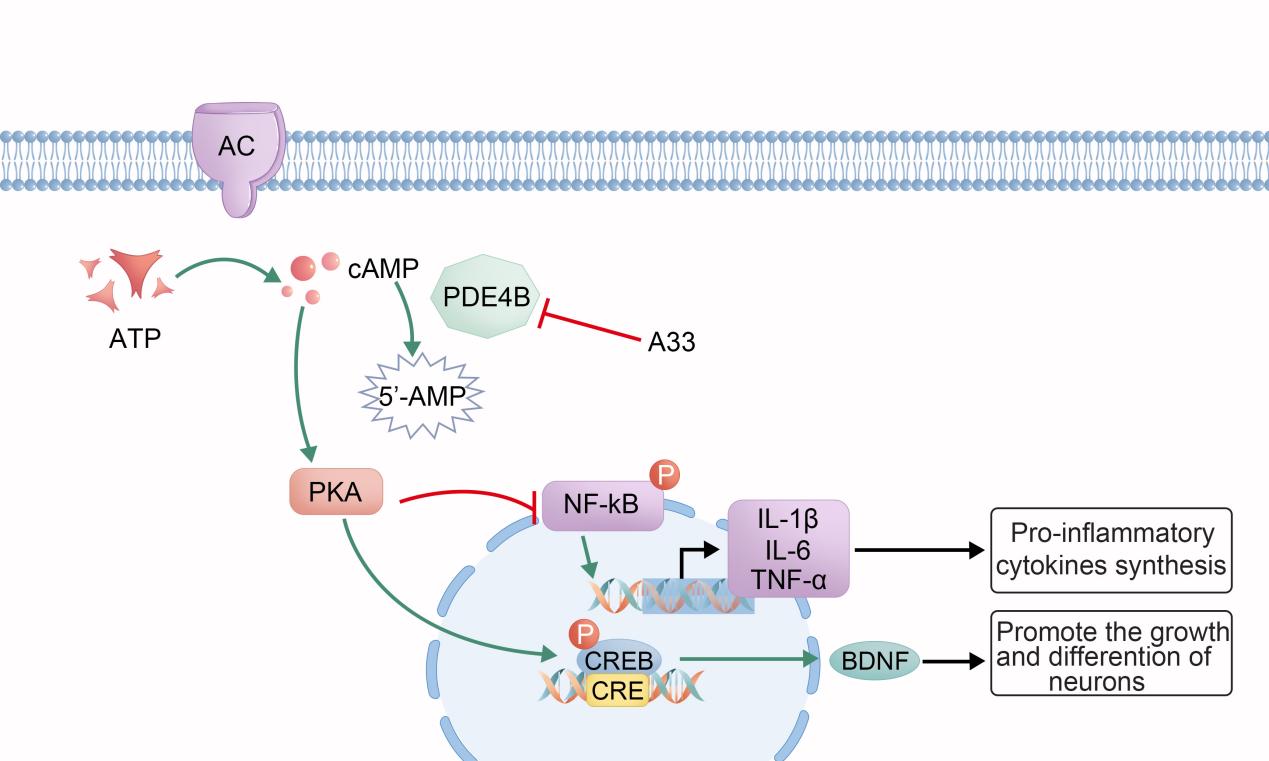


**Sup Fig. 4.** Schematic illustration for intracellular signaling mechanisms of the A33 actions in the treatment of alcoholic dementia. Adenosine triphosphatase (ATP) generates cyclic adenosine monophosphate (cAMP) under the action of adenylate cyclase (AC). Cyclic AMP, as a second messenger in the brain, binds to PKA and promotes its phosphorylation, which in turn promotes the phosphorylation and activation of CREB and increases the production of BDNF. Phosphorylated PKA inhibits the phosphorylation of NF-κB, thereby reducing the production of related inflammatory factors, including IL-1β, IL-6, and TNF-α, and decreasing the occurrence of inflammation. Under the action of PDE4, cAMP is hydrolyzed to 5’-AMP, leading to the reduction of cAMP contents and the loss of its role as a second messenger in the brain. A33 inhibits PDE4B-induced hydrolysis of cAMP and increases the level of cAMP in the brain, resulting in activation of the downstream signaling pathway, which plays a therapeutic role in AlD.
